# Mizoribine therapy combined with steroids and mizoribine blood concentration monitoring for idiopathic membranous nephropathy with steroid-resistant nephrotic syndrome

**DOI:** 10.1007/s10157-016-1340-2

**Published:** 2016-10-25

**Authors:** Takao Saito, Masayuki Iwano, Koichi Matsumoto, Tetsuya Mitarai, Hitoshi Yokoyama, Noriaki Yorioka, Shinichi Nishi, Ashio Yoshimura, Hiroshi Sato, Satoru Ogahara, Yoshie Sasatomi, Yasufumi Kataoka, Shiro Ueda, Akio Koyama, Shoichi Maruyama, Masaomi Nangaku, Enyu Imai, Seiichi Matsuo, Yasuhiko Tomino, The Refractory Nephrotic Syndrome Study Group

**Affiliations:** 1 Professor Emeritus, Fukuoka University, and Sanko Clinic, 4-9-3 Ropponmatsu, Chuo-ku, Fukuoka, 810-0044 Japan; 2 Division of Nephrology, Department of General Medicine, Faculty of Medical Sciences, University of Fukui, Fukui, Japan; 3 The University Research Center, General Science Institute, School of Medicine, Nihon University, Tokyo, Japan; 4 Department of Nephrology and Blood Purification, Saitama Medical Center, Saitama Medical University, Kawagoe, Japan; 5 Division of Nephrology, Kanazawa Medical University School of Medicine, Ishikawa, Japan; 6 Hiroshima Kidney Organization, Hiroshima, Japan; 7 Division of Nephrology and Kidney Center, Kobe University Graduate School of Medicine, Kobe, Japan; 8 Division of Nephrology, Department of Internal Medicine, Showa University Fujigaoka Hospital, Yokohama, Japan; 9 Division of Nephrology, Tohoku University Graduate School of Medicine, Sendai, Japan; 10 Division of Nephrology and Rheumatology, Faculty of Medicine, Fukuoka University, Fukuoka, Japan; 11 Department of Pharmaceutical Care and Health Sciences, Faculty of Pharmaceutical Sciences, Fukuoka University, Fukuoka, Japan; 12 Ueda Clinic, Chiba, Japan; 13 Professor Emeritus, Tsukuba University, Ibaraki, Japan; 14 Department of Nephrology, Nagoya University Graduate School of Medicine, Nagoya, Japan; 15 Division of Nephrology and Endocrinology, University of Tokyo School of Medicine, Tokyo, Japan; 16 Nakayamadera Imai Clinic, Hyogo, Japan; 17 Professor Emeritus, Juntendo University, Tokyo, Japan

**Keywords:** Mizoribine, Idiopathic membranous nephropathy, Steroid-resistant nephrotic syndrome, Once-a-day administration, Estimated serum concentration curve

## Abstract

**Background:**

We designed a prospective and randomized trial of mizoribine (MZR) therapy combined with prednisolone (PSL) for idiopathic membranous nephropathy (IMN) with steroid-resistant nephrotic syndrome (SRNS).

**Methods:**

Patients with IMN were divided into 2 groups, and MZR combined with PSL was administered for 2 years. PSL was initially prescribed at 40 mg/day and tapered. MZR was given once-a-day at 150 mg and 3-times-a-day at 50 mg each to groups 1 and 2. Serum MZR concentrations from 0 to 4 h after administration were examined within one month of treatment. The concentration curve and peak serum level (*C*_max_) of MZR were estimated by the population pharmacokinetic (PPK) parameters of MZR.

**Results:**

At 2 years, 10 of 19 patients (52.6 %) in group 1 and 7 of 18 patients (38.9 %) in group 2 achieved complete remission (CR). The time-to-remission curve using the Kaplan–Meier technique revealed an increase in the cumulative CR rate in group 1, but no significant difference between the groups. Meanwhile, there was a significant difference in *C*_max_ between groups 1 and 2 (mean ± SD: 1.20 ± 0.52 vs. 0.76 ± 0.39 μg/mL, *p* = 0.04), and *C*_max_ levels in CR cases were significantly higher than those in non-CR cases. Receiver operating characteristic analysis showed that *C*_max_ more than 1.1 µg/mL was necessary for CR in once-a-day administration.

**Conclusion:**

Administration of MZR once a day is useful when combined with PSL for treatment of IMN with SRNS. In addition, it is important to assay the serum concentration of MZR and to determine *C*_max_, and more than 1.1 µg/mL of *C*_max_ is necessary for CR.

## Introduction

Idiopathic membranous nephropathy (IMN) is the most representative disease associated with steroid-resistant nephrotic syndrome (SRNS) in adults. In our cohort study of 1000 cases in Japan, the overall renal survival rate for patients with IMN, in which end-stage renal disease (ESRD) was the end point, was 95.8, 90.3, 81.1 and 60.5 % at 5, 10, 15 and 20 years after onset, respectively, and the prognosis was significantly improved by reduction of proteinuria [1]. Accordingly, the primary aims of treatment are to induce a lasting reduction in proteinuria. Although a combination of steroids and immunosuppressants, e.g., alkylating agents [2] and calcineurin inhibitors [3], has been recommended by some guidelines for this purpose, the harmful side effects preclude long-term use of these medicines. On the other hand, several studies have shown that a combination of steroids and mizoribine (MZR), one of the purine metabolism inhibitors, is effective in patients with nephrotic syndrome [4–7] as well as transplant recipients without any serious adverse reactions [8]. MZR is an imidazole nucleotide that exerts selective inhibitory effects on inosine-5-monophosphate dehydrogenase, an enzyme in the de-novo purine nucleoside synthesis system [9, 10], which are very similar to those of mycophenolate mofetil (MMF) and result in suppression of T and B lymphocyte proliferation. Moreover, a recent study has suggested that MZR directly prevents podocyte injury and preserves nephrin structure, leading to a reduction of urinary protein [11]. In fact, this mechanism may clinically reduce the incidence of severe adverse drug reactions. Accordingly, in Japan, MZR is approved as an insurance application medicine for SRNS, and administration at 50 mg 3-times-a-day combined with steroids is recommended. However, it has been pointed out that 3-times-a-day administration cannot provide a serum concentration of MZR sufficiently effective for remission, and that once-a-day administration is more advantageous [12, 13].

To investigate this issue, we designed a prospective, open-label, randomized trial to compare the effect of once-a-day administration of MZR with that of conventional 3-times-a-day administration for IMN with associated SRNS. In addition, the serum concentration curve and the peak serum level (*C*_max_) of MZR after administration, which may reflect the efficacy of MZR, were estimated using serum MZR concentrations assayed at several time points. These estimations were based on a population pharmacokinetic (PPK) analysis of MZR in patients with kidney diseases at Fukuoka University Hospital using a nonlinear mixed effects model (NONMEM) program.

## Methods

This study was entered in the University Hospital Medical Information Network-Clinical Trials Registry (UMIN-CTR) under trial identification No. UMIN C000000368.

### Patients

SRNS patients (age 16–75 years) with IMN diagnosed by renal biopsy were enrolled through computerized registration from kidney centers in Japan between 2004 and 2007. Membranous nephropathy secondary to systemic diseases, e.g., diabetic nephropathy and collagen diseases, were excluded at registration. Nephrotic syndrome (NS) was defined according to the standard criteria used in Japan [1]: (1) urine protein (UP) excretion >3.5 g/day, (2) serum albumin <3.0 g/dL or serum total protein <6.0 g/dL, (3) presence of edema, and (4) total cholesterol >250 mg/dL. At least the first and second criteria were necessary for the diagnosis. SRNS was determined when patients did not achieve complete remission (CR) or incomplete remission (ICR) 1 (as described in the “Clinical assessment” section) after 4 weeks of prednisolone (PSL) therapy at 40–60 mg/day. The inclusion and exclusion criteria are listed in Table 1.

**Table 1:** Inclusion and exclusion criteria

Inclusion criteria
Age between 16 and 75 years
UP ≧3.5 g/day and serum albumin level ≦3.0 g/dL
PSL treatment alone for >4 weeks did not decrease UP to <1 g/day
Membranous nephropathy was diagnosed by renal biopsy
No history of treatment with MZR before registration
Informed consent form signed voluntarily by the participant
Exclusion criteria
Patients with creatinine clearance <50 mL/min or serum creatinine >2 mg/dL
Patients with a history of severe hypersensitive reaction to MZR
Patients previously treated with MZR
Patients with a white blood cell count of <3000/mm^3^ in peripheral blood
Patients currently pregnant, suspected to be pregnant, or nursing
Patients with any severe complication
Patients with any severe bacterial, fungal, or viral infection
Patients determined to be inappropriate for participation in the study by an investigator

*UP* urine protein, *PSL* prednisolone, *MZR* mizoribine

Renal histology was assessed according to the following 5 parameters: presence of global sclerosis and segmental sclerosis in glomeruli, severity of tubulointerstitial changes, occurrence of vascular lesions, and ultrastructural stage of glomerular lesions according to the criteria of Ehrenreich and Churg [14]. These changes were estimated semiquantitatively, as we have previously reported [1], and compared between the groups.

### Trial design

Patients were divided prospectively and randomly into 2 groups (groups 1 and 2). Combined administration of PSL and MZR was continued for 24 months. PSL was initially prescribed at 40 mg/day and tapered gradually to <10 mg/day by 48 weeks. In group 1, MZR was given orally once-a-day after breakfast at 150 mg. In group 2, MZR was given 3-times-a-day after meals at 50 mg each. Other agents, including antihypertensive, antidyslipidemic, antiplatelet and anticoagulant drugs, were allowed unless their combination with MZR was contraindicated. Biochemical data, including total protein, albumin, urea nitrogen, creatinine, uric acid, total cholesterol, aspartate aminotransaminase (AST) and alanine aminotransferase (ALT) in serum, and 24-h UP, were assayed at 0, 3, 6, 12 and 24 months.

### Clinical assessment

Clinical assessment of treatment outcomes was done on the basis of changes in proteinuria and renal function, partly modified from the previous criteria used in Japan [1]. Briefly, CR was defined as UP <0.3 g/day. ICR was defined as the resolution of NS but with continuing overt proteinuria, and was divided into 2 grades: ICR1 and ICR2 for UP values of 0.3–0.99 and 1.0–3.5 g/day, respectively. No response (NR) was defined as the persistence of NS. Since, in a previous study, patients with ICR1 had shown a favorable prognosis almost equal to CR [1], we considered CR + ICR1 as remission. For renal function, 3 categories were defined on the basis of serum creatinine concentration: (1) normal renal function <1.5 mg/dL, (2) renal insufficiency 1.5–3.0 mg/dL, and (3) end-stage renal disease >3.0 mg/dL.

### MZR concentration assay

For monitoring of MZR levels in 22 patients (12 in group 1 and 10 in group 2), serum MZR concentrations were assayed from 0 to 4 h of administration (*C*_0–4_) within the first month of treatment using high-performance liquid chromatography (HPLC) according to the method of Hosotsubo et al. [15]. The MZR quantification limit was 0.05 μg/mL in serum.

### PPK analysis in patients with kidney disease

Using serum MZR concentration data for a total of 304 time points in 64 adult patients with various kidney diseases assayed previously at Fukuoka University Hospital, PPK parameters and their inter-individual variations were estimated by NONMEM program according to the estimation method employed previously in studies of healthy male volunteers [16] and adult renal transplant recipients [17].

The one-compartment model with first-order absorption was parameterized in terms of absorption lag time (ALAG), absorption rate constant (KA), the apparent volume of distribution (V/F), and oral clearance (CL/F). The predicted population mean V/F and CL/F of MZR were modeled using the following equations:

$$CL/F\text{ = }\theta_{1} \times {\text{CLcr }}\left( {\text{L/h}} \right)$$

$$V/F = \theta_{ 2} \times {\text{WT}}\text{ }(L)$$

### Estimated serum MZR concentration in IMN patients

The serum MZR concentration curves and maximum concentrations (*C*_max_) for 22 patients in groups 1 and 2 were estimated simultaneously using assayed *C*_0–4_ by Bayesian inference based on the above PPK parameters. The detailed methods were described in previous papers [16, 17]. The data were compared between each administration group of IMN patients.

### Statistical analysis

Values are given as mean ± SD or median (interquartile range). Differences in clinical characteristics between the 2 groups were evaluated using Student’s *t* and Mann–Whitney *U* tests for continuous variables and Fisher’s exact test for categorical variables. The incidence of remission (CR + ICR1) or CR was compared using Fisher’s exact test. Time-to-remission or CR curves for the 2 therapy groups were estimated using the Kaplan–Meier technique, and the curves were compared using the log-rank test. Receiver operating characteristic (ROC) curve analysis was used to test the prognostic value of the serum MZR concentration expressed as *C*_max_ and to determine the optimum cutoff point for prediction of CR.

All statistical analyses were performed using SPSS for Windows version 22.0 (SPSS Japan Inc., Tokyo, Japan).

## Results

**Fig. 1:**
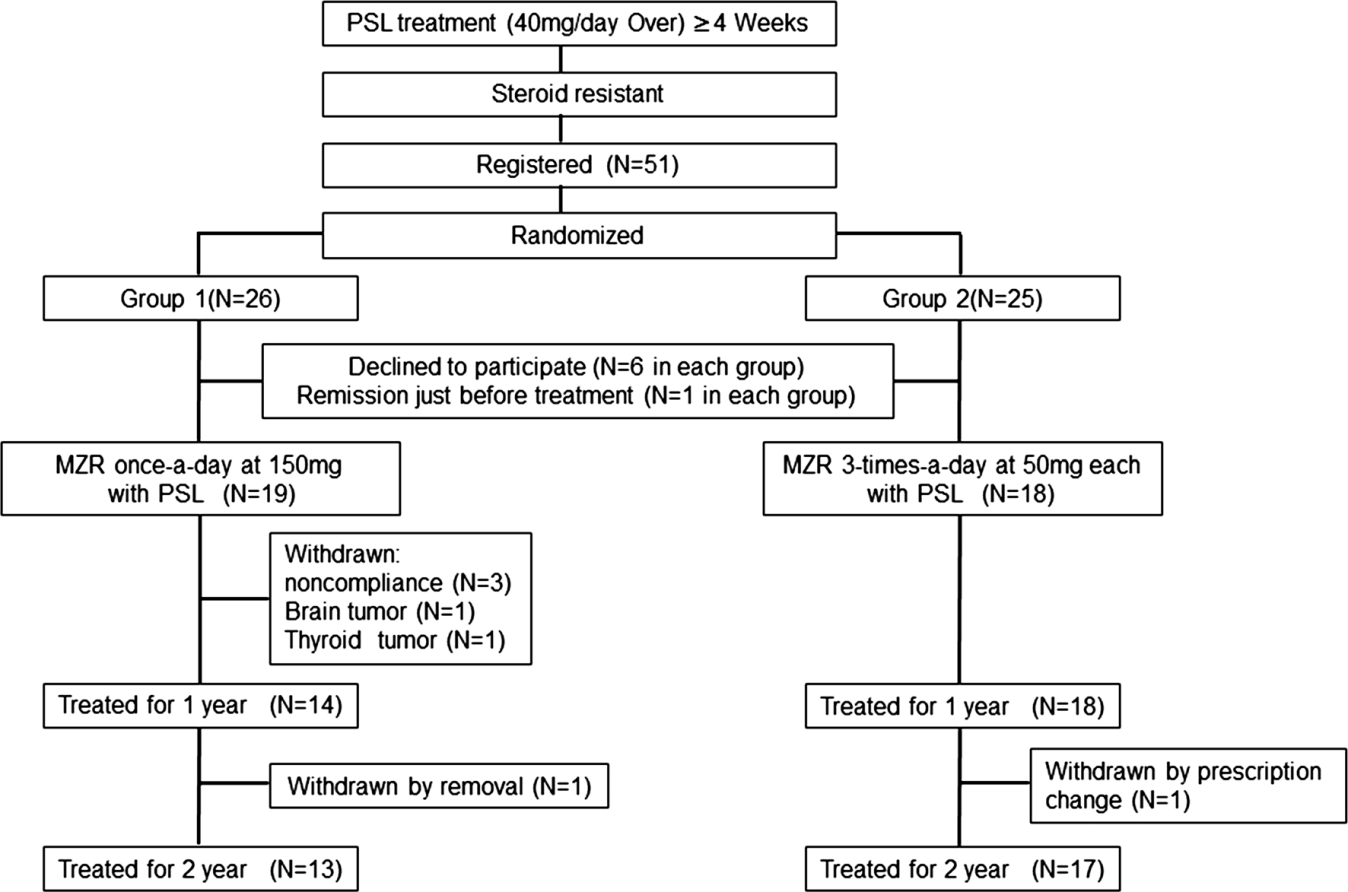
Flowchart of the study design: enrollment of patients and treatment assignment

A flowchart of the study design, including patient enrollment and treatment assignment, is shown in Fig. 1.

### Patients

Fifty-one patients at 23 kidney centers in Japan were registered according to the inclusion criteria, between April 2004 and December 2007, and 26 and 25 patients were randomly enrolled in the once-a-day (group 1) and 3-times-a-day (group 2) administration groups, respectively. However, 6 patients in each group declined to participate in this study before MZR treatment. Moreover, one patient in each group showed urine protein under 1 g/day just before MZR treatment was withdrawn from this study. Consequently, 19 and 18 patients were treated with PSL and MZR in groups 1 and 2, respectively. The baseline clinical characteristics of all patients are summarized in Table 2. The data were at 4 weeks of PSL-alone treatment and just before PSL and MZR combined therapy. There were no significant inter-group differences in any of the characteristics. Furthermore, none of 5 renal histology parameters estimated semiquantitatively showed any significant differences between the groups (data not shown).

**Table 2:** Baseline characteristics of patients with idiopathic membranous nephropathy

Characteristic	Group 1 (*n* = 19)	Group 2 (*n* = 18)	*p*
Gender (male/female)	15:4	14:4	0.93
Age (years)	60 (35–70)	60 (43–74)	0.75
Urine protein (g/day)	3.7 (1.0–7.5)	3.3 (1.3–7.1)	0.80
Serum levels			
Urea nitrogen (mg/dL)	14.5 (7.0–23.7)	15.1 (7.0–29.0)	0.81
Creatinine (mg/dL)	0.8 (0.5–1.3)	0.9 (0.6–1.4)	0.37
Uric acid (mg/dL)	5.7 (3.7–8.5)	6.4 (4.4–9.2)	0.27
Total protein (g/dL)	4.7 (3.9–5.5)	4.7 (3.3–6.2)	0.15
Albumin (g/dL)	2.5 (1.8–3.4)	2.6 (1.0–3.9)	0.59
Total cholesterol (mg/dL)	351 (188–769)	300 (187–390)	0.17

The data were at 4 weeks of PSL-alone treatment and just before PSL and MZR combined therapy

Age and laboratory data are shown as medians (interquartile range)

The *p* values were evaluated by Fisher’s exact test for gender and Mann–Whitney *U* test for the others

### Responses in the once-a-day (group 1) and 3-times-a-day (group 2) MZR administration groups

**Fig. 2:**
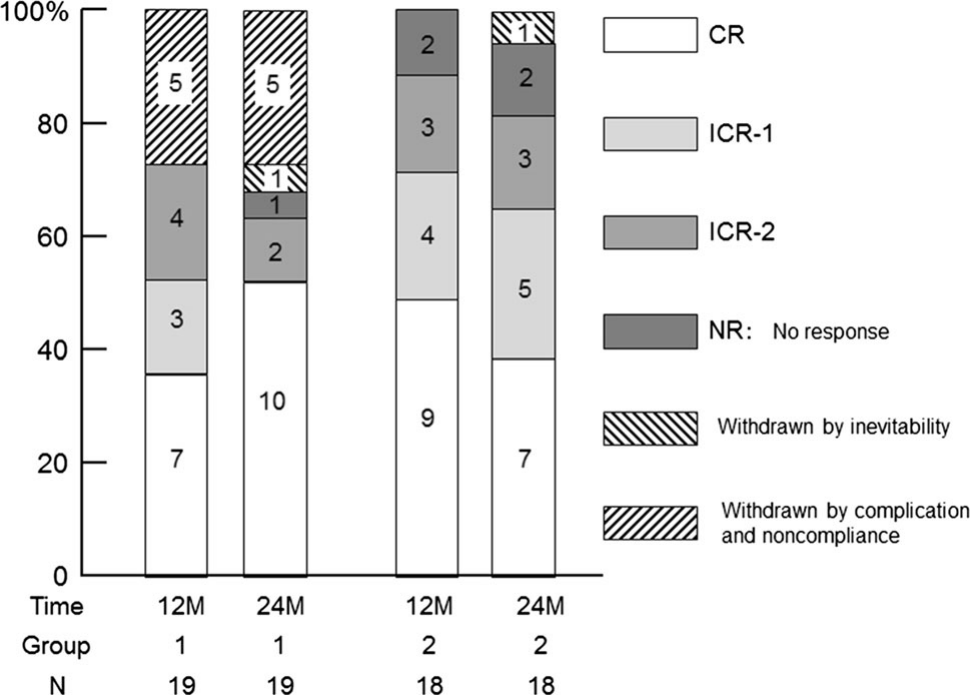
Remission and withdrawal rates of groups 1 and 2 at 12 and 24 months. Patients were divided according to MZR administration frequency—once-a-day (group 1) and 3-times-a-day (group 2)

The responses at one and 2 years of treatment are shown in Fig. 2. Within 1 year of treatment, 9 of 19 patients reached CR in group 1. But one relapsed into ICR2 by 12 months and another was withdrawn by thyroid tumor at 6 months. Accordingly, 7 of 19 patients (36.8 %) in group 1 and 9 of 18 patients (50.0 %) in group 2 achieved CR without relapse at one year. When ICR-1 was added to remission with CR, 10 of 19 patients (52.6 %) in group 1 and 13 of 18 patients (72.2 %) in group 2 achieved remission. Five patients in group 1 were withdrawn because of non-compliance (3 patients), brain tumor (one patient) and thyroid tumor (one patient). In the intention-to-treat analysis, these results did not reveal any significant difference between the groups.

By 2 years, 3 more patients in group 1 reached CR from ICR, and finally 10 patients achieved CR and no patients relapsed, although two ICR2 patients were deteriorated to nephrotic range and withdrawn by removal, respectively. In group 2, one patient reached CR from ICR1, but 3 CR patients relapsed to ICR1 (one patient) and ICR2 (2 patients). On the other hand, two ICR2 patients at 1 year was improved to ICR1 and withdrawn by prescription change, respectively.

As shown in Fig. 1, 13 of 19 patients and 17 of 18 patients completely received MZR treatment combined with PSL for 2 years. With 2 years of treatment, 10 of 19 patients (52.6 %) in group 1 and 7 of 18 patients (38.9 %) in group 2 achieved CR without relapse, and 12 of 19 patients (63.2 %) in group 1 and 12 of 18 patients (66.7 %) in group 2 achieved remission. Accordingly, in the intention-to-treat analysis, these results did not yield a significant difference between the groups.

**Fig. 3:**
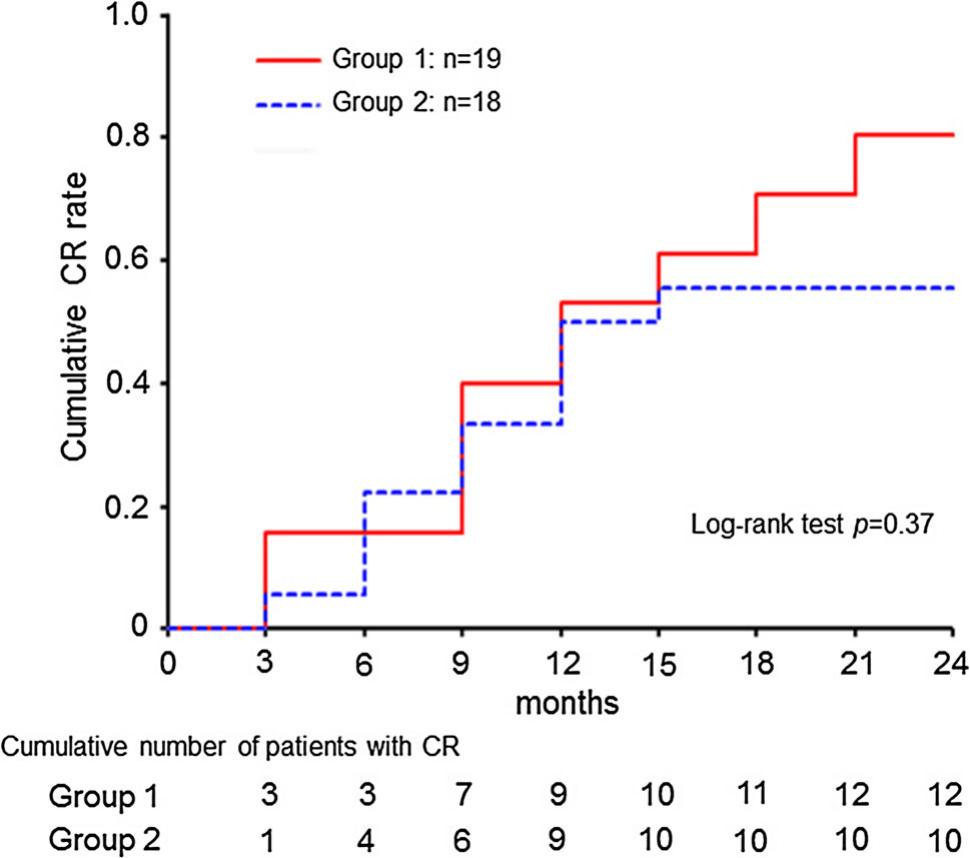
Probability of cumulative complete remission (CR) for patients treated with PSL and MZR by Kaplan–Meier analysis. Cumulative number of CR includes relapse cases. Group 1 showed a slightly higher rate of CR but there was no significant difference between groups 1 and 2

Kaplan–Meier analysis of the time-to-remission curves revealed an increase in the cumulative CR rate in group 1 (Fig. 3), but log-rank test demonstrated no significant inter-group difference.

### Other clinical and laboratory findings

No patients in either group showed serum urea nitrogen or creatinine levels reflecting abnormal renal function. Low total protein and albumin and high total cholesterol concentrations in serum were normalized as remission of NS was achieved. There were no significant differences in these parameters between groups 1 and 2 during treatment. Serum uric acid levels, which are sometimes reported to be elevated in patients receiving MZR, were slightly increased in group 1 during treatment but were not significantly higher than those in group 2 (mean ± SD 7.52 ± 1.31 vs. 6.68 ± 1.45 mg/dL at 2 years of MZR treatment, not significant). Gout was not recognized in any of the patients. AST and ALT levels increased temporarily in some patients in both groups, but were finally normalized, and no patients had serious liver disease.

As described above, two patients in group 1 had to be withdrawn due to development of brain and thyroid tumors, respectively. However, these were accidental complications, and no adverse event related to the use of MZR occurred.

### PPK parameters of MZR

Table 3 shows the data of 64 adult patients with kidney disease for PPK analysis. PPK parameters and their inter-individual variations were estimated by NONMEM analysis and compared with those in previous studies [16, 17] (Table 4). The mean values of ALAG, KA, V/F, and CL/F were 0.675 h, 0.723 h^−1^, 0.744 × WT L, and 2.1 × CLcr × 60/1000 L/h, respectively. The values of PPK parameters in adult patients with kidney disease were almost the same as those in healthy male volunteers [16] and adult renal transplant recipients [17].

**Table 3:** The data of adult patients with kidney disease for PPK analysis

(a) Demographic data of patients used in PPK analysis (*n* = 64)										
Gender (male/female) 31:33	Mean	SD	Mini	Median	Max					
Age (years)	53.8	14.9	19.0	54.5	80.0					
Body weight: WT (kg)	57.0	12.4	27.8	56.7	93.6					
Serum creatinine (mg/dL)	1.1	0.6	0.5	0.9	3.5					
Creatinine clearance: CLcr (mL/min)	71.9	41.1	8.4	67.2	269.9					

(b) Sampling times and numbers of points for PPK analysis										
Time (h)	0	1	2	3	4	6	8	12	24	Total
Points	56	39	59	46	55	25	3	13	8	304

Disease (cases): membranous nephropathy (26), focal segmental glomerulosclerosis (5), IgA nephropathy (7), minimal change nephrotic syndrome (2), Henoch–Schoenlein purpura nephritis (1), kidney transplantation (7), SLE (12), dermatomyositis (2), rheumatoid arthritis (2)

**Table 4:** Comparison of PPK parameters of mizoribine estimated with NONMEM analysis

	Adult patients with kidney disease	Renal transplant recipients^a^	Healthy male volunteers^b^
No. of subjects	64	114	36
Samples	315	449	446
ALAG (h)	0.675	0.581	0.349
KA (h^−1^)	0.723	0.983	0.838
V/F (L)	0.744 × WT	0.558 × WT	0.834 × WT
CL/F (mL/min)	2.10 × CLcr	1.80 × CLcr	1.93 × CLcr

*ALAG* absorption lag time, *KA* absorption rate constant, *V/F* apparent volume of distribution, *CL/F* oral clearance, *WT* body weight, *CLcr* creatinine clearance

The parameters in adult patients with kidney disease are compared with those in adult transplantation recipients^a^ and healthy male volunteers^b^ reported previously

### Estimated serum MZR concentration curves in IMN patients

**Fig. 4:**
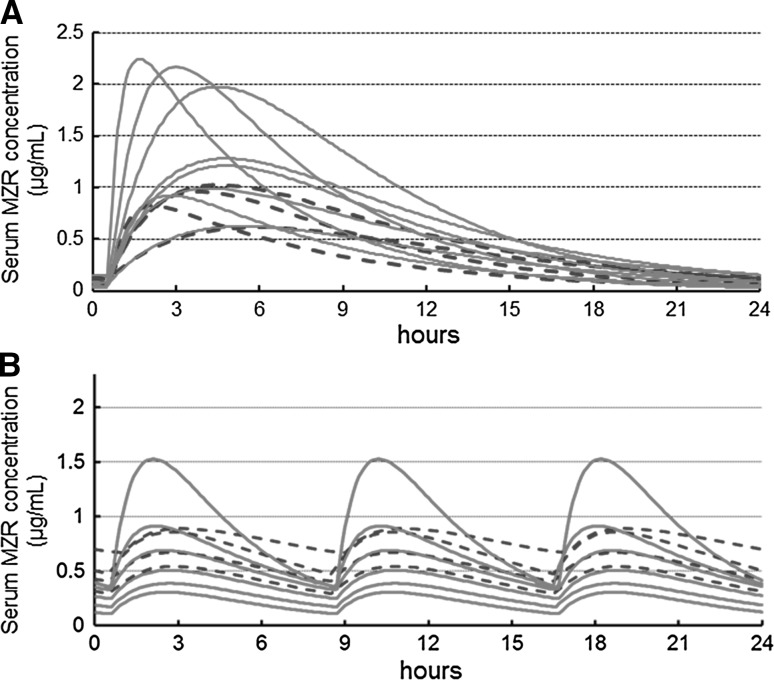
Serum MZR concentration by Bayesian inference based on the PPK parameters following **a** once-a-day and **b** 3-times-a-day administrations. *Solid and dashed curves* are those of CR cases and the others, respectively

Serum MZR concentration curves and *C*_max_ of 12 patients (8 with CR and 4 with non-CR) given MZR once a day and 10 patients (6 with CR and 4 with non-CR) given MZR 3-times-a-day were estimated simultaneously by Bayesian inference based on the PPK parameters described in Table 4 (Fig. 4). The values of assayed *C*_0–4_ and estimated *C*_max_ in 22 patients are summarized in Table 5. *C*_2–4_ were significantly correlated with *C*_max_, and *C*_3_ in particular showed an approximate value of *C*_max_. There was significant difference in *C*_max_ between the two administration protocols (mean ± SD 1.20 ± 0.52 vs. 0.76 ± 0.39 μg/mL, *p* = 0.04). All patients with a *C*_max_ of >1.1 μg/mL, most of whom received the once-a-day regimen, achieved CR, although some with a lower concentration also achieved CR in both groups, and there was no significant difference between CR and non-CR cases (Fig. 4).

**Table 5:** The relations between assayed *C*_0–4_ and estimated *C*_max_ for 22 patients

	Mean ± SD (μg/mL)	Pearson’s correlation coefficient*	p*
*C*_0_	0.045 ± 0.076	−0.319	ns
*C*_1_	0.225 ± 0.202	−0.009	ns
*C*_2_	0.678 ± 0.414	0.857	<0.01
*C*_3_	0.991 ± 0.556	0.984	<0.01
*C*_4_	1.057 ± 0.609	0.962	<0.01
*C*_max_	0.998 ± 0.506	–	–

*ns* not significance

* Between each assayed serum concentration and estimated *C*_max_

**Fig. 5:**
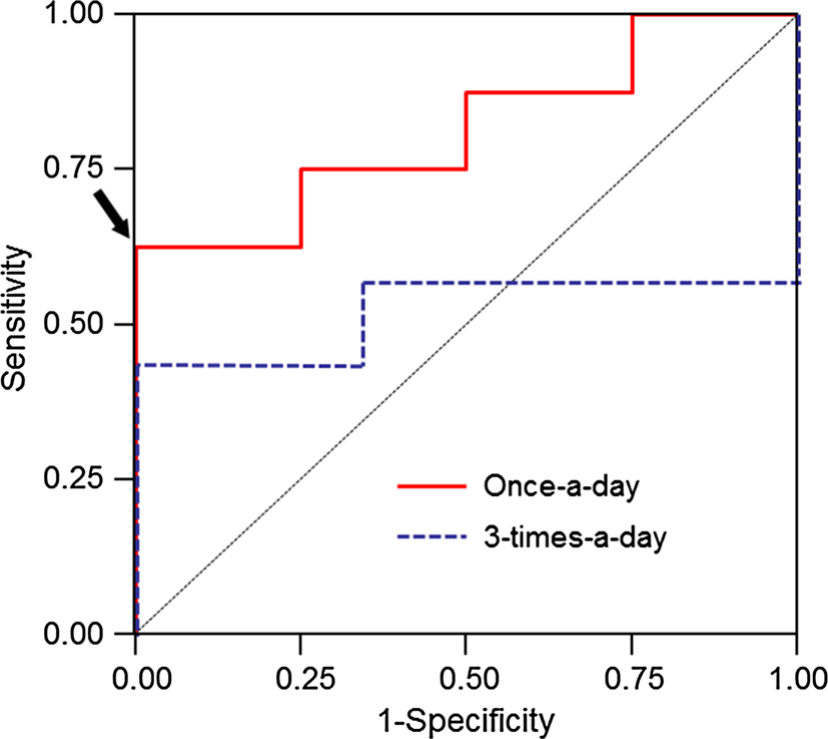
Receiver operator characteristic (ROC) curves for serum MZR concentration in once-a-day and 3-times-a-day administrations. The optimal cutoff level of *C*_max_ for CR was determined to be 1.1 μg/mL (sensitivity 0.625, specificity 1.000) in once-a-day administration (*arrow*)

ROC curves were drawn to detect the optimum cutoff level of *C*_max_ for CR in once-a-day and 3-times-a-day administrations, respectively (Fig. 5). The area under ROC curves were 0.813 ± 0.126 (95 % CI 0.565–1.000) in once-a-day administration and 0.524 ± 0.184 (95 % CI 0.163–0.885) in 3-times-a-day administration. From these results, the optimum cutoff point for *C*_max_ was determined to be 1.1 μg/mL (sensitivity 0.625, specificity 1.000) in once-a-day administration but not in 3-times-a-day administration.

## Discussion

Combined administration of immunosuppressants with steroids has been reported to be useful for the treatment of IMN with associated SRNS. In the KDIGO (Kidney Disease: Improving Global Outcomes) Clinical and Practice Guideline published in 2012 [2], the initial use of cyclophosphamide (CPA), an alkylating agent, with steroids was recommended on the basis of existing evidence accumulated from many randomized controlled trials conducted over the last few decades. However, a cohort study of 1000 cases conducted in Japan did not demonstrate any superiority of combined therapy with steroids and CPA over steroid monotherapy [1]. Moreover, the use of CPA has been associated with adverse events such as neoplasia, agranulocytosis and viral hepatitis, which can sometimes be fatal. On the other hand, cyclosporine (CyA), a calcineurin inhibitor, has been used as an effective agent for IMN with SRNS [18–20] and its combination with steroids is recommended in some guidelines for SRNS therapy [2, 3]. However, CyA also has serious adverse effects such as nephrotoxicity and hypertension, and is not suitable for long-term therapy. From this viewpoint, MZR is known not to have serious adverse events and to be beneficial when administered over a long period [4–7].

MZR is an imidazole nucleotide that inhibits purine synthesis and T and B lymphocyte proliferations [9, 10]. These actions are very similar to those of MMF, but less myelosuppressive and less hepatotoxic [21, 22]. In the present trial, two patients had to be withdrawn due to brain tumor and thyroid tumor, respectively, but these were accidental events and any serious adverse effects induced by MZR were not found. Although it was assumed that hyperuricemia would occur, in view of the inhibitory effect of MZR on purine synthesis, no severe hyperlipidemia or gout was recognized. Moreover, no marked liver dysfunction was recorded. Thus, in agreement with other studies [4–7], our results indicate that MZR is a safe immunosuppressant for long-term use.

MZR has been approved for administration at 50 mg 3-times-a-day with steroids for treatment of nephrotic syndrome by health insurance system in Japan. On the other hand, it has been suggested that once-a-day administration can achieve a higher serum MZR concentration and be more beneficial for intensive immunosuppression in patients with systemic lupus erythematosus [23–25] or renal transplantation [21, 26]. Therefore, in the present study, we compared the once-a-day and 3-times-a-day administration protocols. We found that the cumulative CR ratio in the once-a-day group was increased during 2 years of treatment, although Kaplan–Meier analysis indicated no significant difference between the two administration protocols (Fig. 3). In addition, the remission (CR + ICR) rates of >50 % in both groups were equal to that in a PSL and MZR-treated group (55 %) and much better than that in a PSL-alone group (17 %) in a previous postmarketing randomized controlled trial [5].

To clarify the relationship between MZR dose regimens and response, we estimated the serum MZR concentration curve and *C*_max_ based on PPK analysis and verified that the levels of these parameters were related to the MZR dosage schedule and reflected remission of NS. MZR is absorbed from the gastrointestinal tract after oral administration, and the unchanged drug is excreted predominantly into the urine. The PPK of MZR is well described by a simple one-compartment model with first-order absorption. Accordingly, the MZR concentration curve and C_max_ were previously estimated based on PPK analysis in healthy subjects [16], renal transplant recipients [17] and pediatric patients with kidney disease [27]. However, this is the first time that the MZR concentration curve and *C*_max_ based on PPK analysis in adult patients with kidney disease were applied for the therapeutic response in NS. Once-a-day administration of MZR induced significantly higher *C*_max_ levels than 3-times-a-day administration, although individual *C*_max_ levels were widely distributed (Fig. 4). Actually, several studies suggest that the bioavailability of MZR in each patient is influenced by the intestinal absorption based on polymorphism of concentrative nucleoside transporter 1 gene [28, 29] and salt intake [30]. Therefore, the wide distribution of *C*_max_ was already reported in transplanted recipients [17] and pediatric patients [27].

Meanwhile, all patients with a *C*_max_ of >1.1 μg/mL achieved CR as well as some with lower *C*_max_ values in both administration groups. In addition, ROC analysis showed that the optimum *C*_max_ cutoff point for CR was determined to be 1.1 μg/mL in once-a-day administration alone, and suggested the favorable therapeutic effect of once-a-day administration.

Since our previous cohort study of 1000 cases in Japan [1] found that IMN with NS was frequently responsive to steroid monotherapy, it is possible that CR would be achieved even in patients with a low *C*_max_ of MZR if steroids are given. On the other hand, several recent studies have shown that MZR enhances steroid receptor activity via 14-3-3 proteins corresponding to a blood MZR level [31] and that the clinical efficacy of MZR may be dependent on *C*_max_ [32, 33]. Therefore, a high *C*_max_ level may be necessary to achieve remission in nephrotic syndrome. From this viewpoint, once-a-day administration and increase of MZR dose to reach the cutoff level while monitoring *C*_max_ may be favorable if any side effects are not recognized. When it is difficult to estimate *C*_max_ practically, *C*_3_ can be used in place of *C*_max_ because *C*_3_ is an approximate value of *C*_max_ (Table 5). However, our trial was an open multicenter postmarketing study in which the dose of MZR was limited to within 150 mg/day, i.e., the dosage approved by the health insurance system in Japan. Accordingly, to obtain more precise data on the inter-relationship of administration dose with the serum concentration and effectiveness of MZR, further studies with a larger cohort showing a higher dose will be needed.

In conclusion, MZR is a safe and effective immunosuppressive agent. The combination of PSL and MZR will be useful for treatment of IMN with SRNS if once-day-administration of MZR can achieve more than 1.1 µg/mL of *C*_max_. To predict the response of MZR, it is important to assay its serum concentration and to estimate *C*_max_ based on the PPK parameters of MZR.

## Appendix: The following members organized the trial

**Organizer** Takao Saito.

**Protocol committee** Hiroshi Sato, Shinichi Nishi, Tetsuya Mitarai, Koichi Matsumoto, Ashio Yoshimura, Hitoshi Yokoyama, Masayuki Iwano, Noriaki Yorioka, and Takao Saito.

**Assessment committee** Yasuhiko Tomino, Akio Koyama, and Shiro Ueda.

**Statistics committee** Yasufumi Kataoka, Satoru Ogahara, and Yoshie Sasatomi

**Advisory committee** Seiichi Matsuo, Enyu Imai, Masaomi Nangaku, and Shoichi Maruyama

*The following investigators participated in the trial*

Saitama Medical University: Tetsuya Mitarai, Hajime Hasegawa and Koichi Togasawa; Nihon University: Koichi Matsumoto and Takayuki Fujita; Showa University Fujigaoka Hospital: Ashio Yoshimura, Yoshikuni Nagayama and Takahiro Nakayama; The Jikei University: Tetsuya Kawamura and Hideo Okonogi; Tokyo Women’s Medical University: Wako Yumura, Minako Koike, and Masayo Naito; St. Marianna University School of Medicine: Kenjiro Kimura and Yasuda Takashi; Dokkyo Medical University: Toshihiko Ishimitsu and Hidehiko Ono; Toho University: Sonoo Mizuiri; Tokyo Medical University Hachioji Medical Center: Masaharu Yoshida and Masakazu Akashi; Fujita Health University: Satoshi Sugiyama; Nara Medical University: Masayuki Iwano; Kitano Hospital: Eri Muso; Kanazawa University: Hitoshi Yokoyama; Okayama University: Hirofumi Makino and Yohei Maeshima; Hiroshima University: Noriaki Yorioka and Takao Masaki; Shimane University: Takafumi Ito; Fukuoka University: Takao Saito, Satoru Ogahara and Yoshie Sasatomi; Kurume University: Keisuke Kono.
